# Current Status of Fetal Echocardiography Imaging and Fetal Counseling Fellow Training in 29 European Countries

**DOI:** 10.1007/s00246-025-04006-0

**Published:** 2025-09-01

**Authors:** Taisto Sarkola, Anna N. Seale, Andreas Tulzer, Sophie M. Duignan, Agnieszka Grzyb, Giulia Tuo, Ellen Vanhie, Colin J. McMahon

**Affiliations:** 1 Children’s Hospital, University of Helsinki and Helsinki University Hospital, Stenbäckinkatu 9, PO box 281, 00029 Helsinki, Finland; 2 Minerva Foundation Institute for Medical Research, Helsinki, Finland; 3 Institute of Cardiovascular Sciences, University of Birmingham, Birmingham, UK; 4 Heart Unit, Birmingham Women’s and Children’s NHS Foundation Trust, Birmingham, UK; 5 Department of Pediatric Cardiology, Children’s Heart Center Linz, Kepler University Hospital, Linz, Austria; 6 Medical Faculty, Johannes Kepler University Linz, Linz, Austria; 7 Department of Paediatric Cardiology, Children’s Health Ireland at Crumlin, Dublin, Ireland; 8 School of Medicine, University College Dublin, Belfield, Dublin, Ireland; 9 Department of Perinatal Cardiology and Congenital Anomalies, Centre of Postgraduate Medical Education, Warsaw, Poland; 10 Department of Cardiac Surgery, The Children’s Memorial Health Institute, Warsaw, Poland; 11 Pediatric Cardiology and Cardiac Surgery Unit, Surgery Department, IRCCS Instituto Giannina Gaslini, Genoa, Italy; 12 Pediatric Cardiology, Paola Children’s Hospital, Antwerp, Belgium; 13 Fetal Cardiology Department, University Hospital Ghent, Ghent, Belgium

**Keywords:** Fetal echocardiography, Fetal counseling, Training

## Abstract

Limited data exist on the implementation of current fetal cardiology training and practice guidelines, how trainees are assessed, and how trained fetal cardiologists maintain their skills among countries affiliated with the Association of European Paediatric and Congenital Cardiology (AEPC). A structured questionnaire was sent to fetal cardiologists or national delegates from 44 centers in 33 European countries. Responses were obtained from 37 centers in 29 European countries with 31 responses from fetal cardiologists. Fetal echocardiography was equally performed in maternal (18) and pediatric (16) hospitals with median 3 (range 0–6) fetal cardiologists per center and > 4 fetal cardiologists in 13 centers. Core and advanced fetal cardiology training was offered in 17 (46%) and 21 (57%) centers. Advanced training was provided in higher volume centers (19/21). Assessment methods included direct trainee observation, case-based discussions, and participation in multidisciplinary team meetings, with mostly verbal feedback provided. Criteria for independent fetal echocardiography and counseling were based on training duration (range 2–24 months), number of assessments (range 100–1500), and number of counseling abnormal cases (range 40–200) performed under expert supervision, as well as on expert evaluations of trainees based on direct observation and fetal cardiac diagnostic accuracy. Formal certification in fetal cardiology was reported in three centers. Research activity among trained experts was reported among 25 (68%) respondents overall with 19 respondents involved with collaborative research. Trainee research was encouraged but not mandatory in clinical training. Maintenance of expert skills included sufficient clinical activity volume, teaching, and different forms of national and international networking. Fetal cardiology service quality assessments included missed cases discussion in 20 (54%) centers. There is substantial variation in advanced fetal cardiology training practice in Europe suggesting a need for further clarification of training criteria and structure. Trainee assessment is mainly verbal and based on direct observation. There seems to be a need to strengthen the fetal cardiology module in core pediatric cardiology training and to improve quality assessment of the clinical service provided.

## Introduction

Significant improvement in prenatal detection of cardiac abnormalities during routine ultrasound obstetric screening over time has shifted the timing of diagnosis of major congenital heart diseases (CHD) to the prenatal phase. This provided opportunity for improvements in both prenatal care and follow-up, perinatal care planning, as well as parental counseling. The role of fetal cardiology as a subspeciality has then significantly evolved with increasing service demands including high accuracy of fetal cardiac diagnosis mainly based on echocardiography, timely referral for additional multidisciplinary services and investigations needed to assess associated extra-cardiac problems, genetic testing, as well as anticipated prenatal and perinatal care planning. The fetal cardiologist is a key figure in multidisciplinary teamwork and usually takes the lead in communicating diagnosis, prognosis, and details on pre-, peri- and postnatal care to parents. All of this requires a high level of knowledge and skill in fetal cardiac diagnosis, natural history, treatment and management of disease, as well as parental counseling. To address all these aspects, clinical guidelines for fetal echocardiography [1, 2] as well as management and counseling practice [3, 4] have been developed both in North America and Europe, and also on national levels in Europe [5].

Basic fetal cardiology knowledge is included in core pediatric cardiology training but without requirement for independent fetal imaging or counseling skills [6, 7]. However, advanced fetal cardiology training is needed to meet service needs outlined in fetal cardiology practice guidelines [3, 4] and usually commenced as subspeciality training after completed core training. To date, however, there is limited information available on the implementation or current state of fetal cardiology training in core pediatric cardiology or advanced fetal cardiology curricula in Europe or other parts of the world.

We hypothesized that there is marked variation in fetal cardiology training overall, concerning fetal echocardiography, management as well as counseling among centers in Europe. The aim of this survey was then to get an understanding of current fetal cardiology training practice in major European centers, particularly related to: 1. Training practice and training criteria for fetal echocardiography and counseling both at core and advanced levels, 2. to outline how fetal cardiology trainees are assessed and how the training is provided, and 3. to evaluate how trained fetal cardiologists maintain their skills.

## Materials and Methods

In autumn 2024, a structured questionnaire was designed by the fetal cardiology working group council of AEPC to ascertain fetal cardiology training in major European countries providing population-based screening for fetal cardiac abnormalities as a part of routine obstetric anomaly screening, as well as pre-, perinatal and postnatal treatment. The questionnaire included a total of 45 questions with 27 questions allowing for additional qualitative information. Questions were divided into domains of respondent background and center characteristics (13 questions), fetal cardiology core and advanced training (22 questions), and trained fetal cardiologist continuous education and research practice (10 questions). The questionnaire was circulated to pediatric cardiology training centers registered with the AEPC and, in all, sent to fetal cardiologists or national delegates from 44 centers in 33 European countries (https://www.surveymonkey.com/r/3V393K2). We requested that the survey be completed, when possible, by a person with advanced fetal cardiology training and closely involved with fetal cardiology service and knowledge of fetal cardiology training in the respective geographical areas. The questionnaire detailed respondent training and role, fetal cardiology practice in the geographical area, number of trainees in respondent center, details related to requirements and criteria of training, assessment and feedback practice for both core pediatric cardiology and advanced subspeciality fetal cardiology trainees in fetal echocardiography, assessment and feedback practice for both core pediatric cardiology and advanced subspeciality fetal cardiology trainees in fetal counseling, overall teaching design and practice. Additional open-ended questions addressed more detailed information about center fetal cardiology practice and training as well as outlined strengths and weaknesses of the fetal cardiology training provided. Most respondents replied to all questions provided and completed the survey although none of the survey questions were mandatory. The number of missing responses to main questions was generally small, but the proportion of responses to additional open-ended questions varied.

All survey replies were included in analyses. Descriptive statistics were performed for all survey questions and reported as N or median with range, as found appropriate.

As the survey was intended to be disseminated among international peers and collecting anonymous data without active recruitment of patients or staff as participants, the requirement for a full review by the Children’s Health Ireland Research ethics committee was waived. Consent to collect and publish the survey data in a peer-reviewed international journal was obtained from all survey respondents in conjunction with the survey.

## Results

**Fig. 1:**
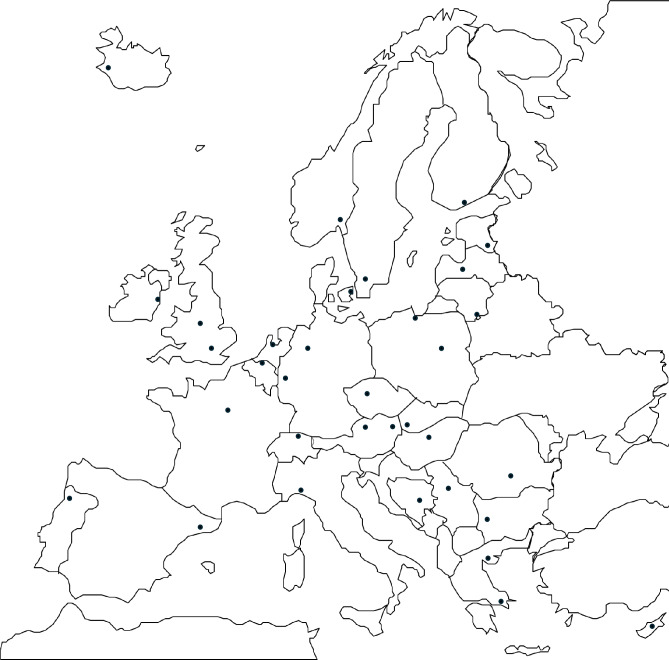
Survey participants geographical distribution in the AEPC fetal cardiology training survey representing 37 centers in 29 European countries

Responses were obtained from 37 centers from 29 European countries with a response rate of 84 and 88 percent, respectively (Fig. 1). No responses were obtained from four centers. More than one response was provided from Austria (2), Germany (2), Greece (3), Hungary (3), Ireland (3), Poland (2), and United Kingdom (2). More than one response was obtained from one center only with results combined in analyses. Responses were obtained from 31 persons with completed training in pediatric cardiology and advanced fetal cardiology, 4 persons with completed training in pediatric cardiology but without formal advanced fetal cardiology training, 1 fetal medicine specialist, and 1 pediatric cardiology trainee. The pediatric cardiology trainee response was combined with other trained expert responses from the same center. Thus, the survey results represent mainly responses from pediatric cardiologists with advanced fetal cardiology training.

### Outline of Survey Respondent European Centers

**Fig. 2:**
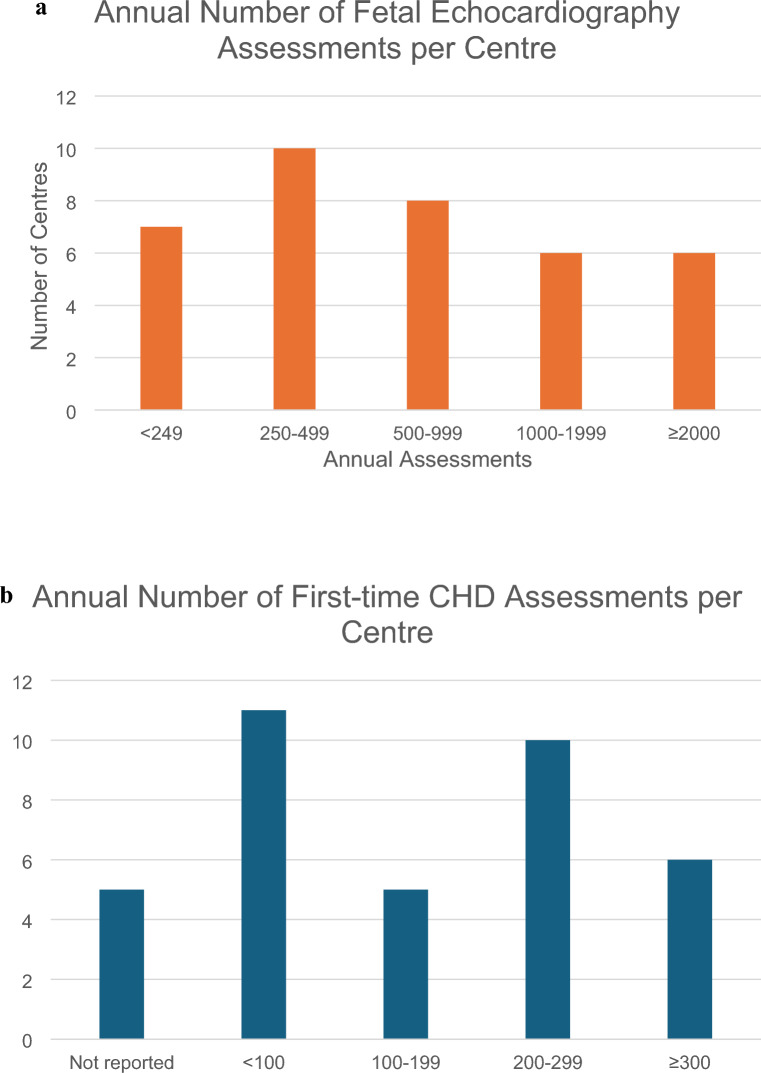
Annual number of fetal echocardiography assessments (**A**) and first-time congenital heart disease assessments (**B**) per center in Europe

**Fig. 3:**
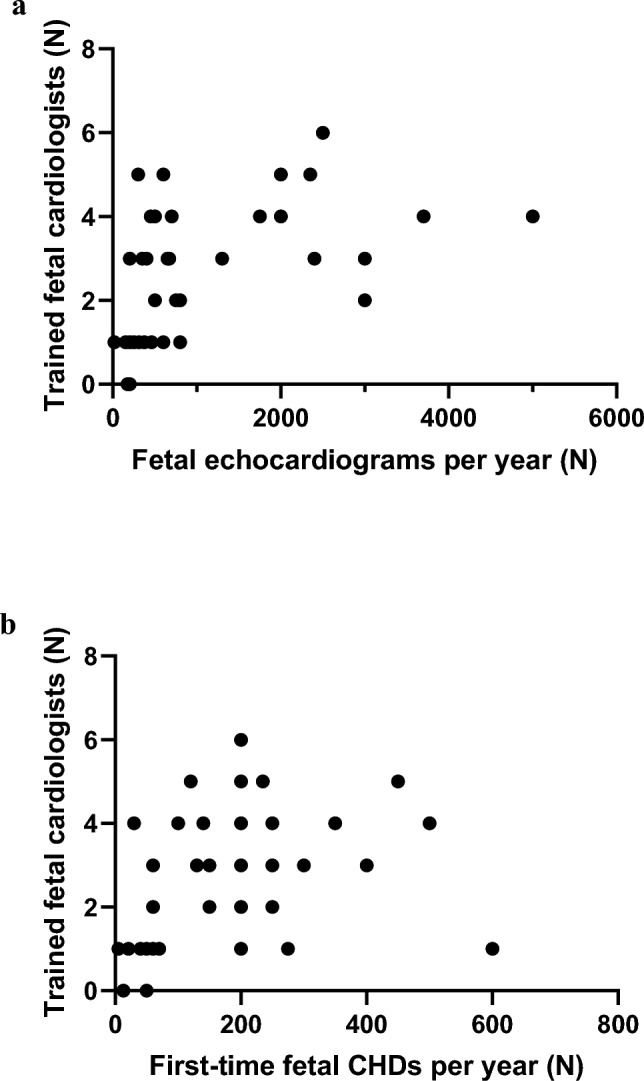
Scatter plots relating total number of fetal echocardiograms per year (**A**; Spearman *r* = 0.56, *p* = 0.0003) and first-time congenital heart disease (CHD) assessments per year (**B**; Spearman *r* = 0.43, *p* = 0.0096) with number of trained fetal cardiologists

Of 37 respondents, 31 represented centers with reportedly dedicated fetal cardiologists. Clinicians interpreting fetal echocardiograms and performing fetal counseling at the respondent’s centers reported the following training backgrounds: combined pediatric and fetal cardiology 27, pediatric cardiology 5, and combined pediatric cardiology and perinatology/fetal medicine specialist 5. The median number of trained fetal cardiologists per center was 3 (range 0–6) with three centers providing fetal cardiology service with personnel lacking formal fetal cardiology training and 13 centers with more than four trained fetal cardiologists. Fetal echocardiographic assessments were performed in fetal medicine or obstetric center outpatient clinics in 18, children’s hospital outpatient clinics in 16, and specialized fetal cardiology units in 3 centers. The reported number of fetal echocardiography assessments per year were more than 2000 in six, 1000–1999 in six, 500–999 in eight, 250–499 in ten and less than 249 in seven centers. The total number of first-time congenital heart disease (CHD) cases assessments per year was 300 or more in six, 200–299 in ten, 100–199 in five, less than 100 in eleven, and not reported in five centers (Fig. 2). There was a significant association between total number of fetal echocardiograms per year, first-time CHD assessments per year, and number of trained fetal cardiologists per center (Fig. 3). A standard fetal echocardiogram imaging protocol was followed in 28 (76%) centers. Among the respondent centers, fetal cardiac interventions were offered in eight centers (Barcelona, Budapest, Genova, Linz, London, Paris, Warsaw, and Bucharest).

### Fetal Cardiology Training in Europe

Fetal cardiology was included as part of core pediatric cardiology training in 17/37 (46%) centers only. Subspeciality advanced fetal cardiology training was overall offered in 21/37 (57%) centers including all 20 centers performing more than 500 fetal echocardiographic studies and assessing more than 100 CHD cases per year, and in the remaining two centers performing 250–499 studies with less than 100 CHD assessments per year. Advanced training was not provided in centers with less than 250 fetal studies per year. Six centers reported following only national, four European/AEPC (2004), six AHA/Canadian, fourteen mainly ISUOG, and nine other advanced fetal cardiology training guidelines.

Advanced level training characteristics are outlined in Table 1. For training longer than 12 months (18 centers), it was specified as 24 months in two responses and 12–24 months in another four responses. For shorter training duration (10 centers), responses were specified as 12 months in one, 6–12 months in one, 6 months in one, 4 months in one, and 2 months in two responses. Screening views (4-chamber, outflow tracts, and 3-vessel view) were recommended first prior to a more detailed fetal echocardiography assessment by 31 respondents.

**Table 1:** Advanced fetal cardiology training characteristics in 37 centers in Europe

Required training duration:
> 12 months (18 centers)
< 12 months (10 centers)
training not provided (5 centers)
duration not specified (4 centers)
Main criteria for trainees to provide independent fetal echocardiography image interpretation and CHD counseling:
1. Completed core pediatric cardiology training (all respondents)
2. Number of fetal echocardiogram assessments performed (range 100–1500) under supervision (20 respondents)
3. Number of abnormal findings fetal CHDs counseled under supervision (range 40–200) (20 respondents)
4. Fetal cardiology certification or approval by senior fetal cardiologist based on observation of practice (20 respondents)
5. Required training duration completed (6 respondents)
Methods of training provided:
1. Trainees observing fetal cardiology consultations (30 responses)
2. Supervised fetal echocardiography assessment and counseling (15 responses),
3. Didactic teaching or formal course on counseling (5 responses)
4. Fellows attending multidisciplinary meetings (2 responses)
Methods of providing feedback to trainees included the following:
1. Hands-on training during echocardiography (26 responses)
2. Feedback on every echocardiogram performed (21 responses)
3. Feedback after echocardiography (10 responses)
4. Feedback on echocardiograms only when requested by trainees (4 responses)
5. Imaging report generated by the trainees (21 responses)
6. Monitoring of imaging diagnosis accuracy in comparison with postnatal imaging or fetal autopsy reports (3 centers)
7. Feedback only in conjunction with training evaluation and feedback discussions (4 responses)
8. Regular reviews of competence progression (5 centers)

When asked to describe center criteria for trainees to independently perform fetal echocardiography assessment and fetal cardiology counseling in the setting of an abnormal fetal echocardiogram/assessment, core pediatric cardiology training was required as a basic training prerequisite by a majority (Table 1). 6 respondents mentioned criteria based on advanced fetal cardiology training duration and 20 respondents included criteria based on total number of fetal echocardiogram assessments performed under supervision (range 100–1500) with abnormal findings (CHDs) counseled under supervision (range 40–200), and logbook of training. Training abroad in a large volume center was required by one respondent from a larger volume center. Fetal cardiology certification or approval by senior fetal cardiologist based on observation of practice was mentioned by 20 respondents. However, no respondent specified clear standard criteria for the approval.

Fetal cardiology counseling teaching was provided by having fellows in training observing fetal cardiology consultations, by observing fellows in training providing fetal cardiology counseling under the supervision of a trained fetal cardiologist, by providing didactic teaching or formal course on counseling, and advanced fellows attending multidisciplinary meetings (Table 1).

An objective measure for assessing the quality of fetal echocardiographic imaging in the center was reported among 9 respondents. Imaging quality was mainly assessed qualitatively by review of images by senior fetal cardiologists (15 respondents). Feedback to the trainees on their fetal echocardiographic imaging quality was provided during and after imaging (Table 1). Fetal echocardiography diagnosis accuracy in comparison with postnatal imaging or fetal autopsy reports was monitored in 3 centers with regular feedback evaluations provided. The fetal echocardiogram report was generated by the trainee in 21 responses. Feedback was provided on every echocardiogram performed by the trainee in 21 responses, and only when requested by the trainee in 4 responses. Feedback was provided for trainees only in conjunction with training evaluation and feedback discussion meetings outside of outpatient clinic visits in 4 responses. Feedback was generally described as verbal discussions prior to and during imaging and counseling, and debriefing after. Fetal counseling was not provided by trainees in 4 responses. Simulation in fetal cardiology training was provided in 3 responses only.

In all, 22 responses were provided on how competence assessment was performed for core and advanced fetal cardiology fellows. These included direct observation, evaluation of fetal images obtained, reports generated and counseling provided by trainees, discussing cases to evaluate knowledge of fetal hemodynamics and perinatal care planning, using a logbook, performing practical and theoretical examinations to assess competence, providing regular feedback as well as performing educational supervisor performance reviews, and finally by seeking agreement among team members on trainee imaging and counseling skills, strengths and limitations needing improvement. In most responses, core fellows were not expected to perform/interpret fetal imaging or provide fetal counseling independently. On the contrary, at advanced fellows’ competence level it was expected to meet criteria of independent fetal imaging and interpretation as well as fetal counseling, therapy, and perinatal care planning. Annual reviews of competence progressions were performed for advanced fellows in 5 centers only.

Fetal echocardiographic imaging training was assessed by case-based discussions in 18 responses, and workplace-based assessments in 13 responses. Observed Structured Clinical Examinations (OSCE) were performed in one center only. Exit exams or attending the EACVI exam were reported in one response only. Nine respondents reported using none of the methods listed above. Research was not required as part of advanced subspeciality fetal cardiology training program but encouraged as part of daily activity in 7 responses. A certification for subspeciality fellows in fetal cardiology was provided in three responses only.

### Maintenance of Fetal Cardiologist Skills

A minimum number of fetal echocardiograms and consultations that fetal cardiologists are expected to perform annually to maintain competence were reported in 6 responses (range 20–200). In addition, skills were maintained by international networking with colleagues and experts, by annual attendance and participation in international meetings with fetal cardiology programs and presenting scientific abstracts, by performing international research article peer-reviews, and by participating in and organizing fetal cardiology teaching. Collecting Continuing Medical Education (CME) points was reported by two respondents only. A formal feedback process for missed cases (both false normal and false abnormal fetal cardiac evaluations with significant clinical impact) was included in 20 (54%) responses. Regular multidisciplinary meetings including fetal cardiologists, specialist nurses and midwives, perinatologists and obstetricians, neonatologists and intensivists, surgeons, social workers, psychologists (all as found appropriate) involved in the care of families were arranged in 28 (76%) centers. Fetal cardiology service quality evaluations to improve service were undertaken in 18 (49%) responses. Independent research in fetal cardiology by the center was performed in 25 (68%) centers. 19 respondents were engaged in collaborative fetal cardiology research projects across European centers.

## Discussion

This survey provides an outline of the current fetal cardiology training practice in Europe and shows substantial variability in the volume of fetal cardiology practice among centers, with advanced training provided predominantly in higher volume centers. Fetal cardiology training for core pediatric cardiology fellows was relatively rare. Duration of training and number of fetal assessments and counseling required in advanced training was highly variable. Competence assessments were based mainly on direct observation of imaging quality, accuracy of diagnosis and missed cases assessments as well as direct observation of counseling.

The survey highlights a variance and diversity in fetal cardiology training likely related to significant variability in legislation, funding, and organization of health care systems among European countries. With improved prenatal detection of congenital heart disease and lack of advanced fetal cardiology service in proximity of obstetric screening center, pediatric cardiologists with core level training may be asked to be involved in fetal counseling of a family with a fetus with major CHD. Although fetal cardiology in core pediatric cardiology training is included in guidelines [4, 6, 8] as well as in the AEPC pediatric cardiology exam, according to this survey training was offered for core pediatric cardiology trainees in 46% of centers only. Core fellow training requirements did not include independent fetal echocardiography image interpretation and counseling which, according to the survey, was required for advanced subspeciality trainees. The relatively low proportion of respondents providing fetal cardiology training to core fellows, nevertheless, suggests a need to strengthen fetal cardiology training in the pediatric cardiology core curriculum. However, an understanding of the fetal cardiac ultrasound images, including the interpretation of screening views and the fetal echocardiogram, is important as diagnostic accuracy has a major impact on prenatal and perinatal care planning [9], and should, therefore, be provided by trained fetal cardiologists for service quality reasons.

Current advanced fetal cardiology training guidelines recommend subspeciality training commenced following core training in pediatric cardiology or perinatal medicine. Training should preferably be provided in larger volume fetal cardiology centers allowing for exposure to the variability of diagnostic severity and care planning of rare complex CHDs. The survey results support this. However, there seems to be a lack of consensus on training criteria and requirements for advanced independent fetal cardiology practice. Ideally, training should meet the practice requirements outlined both in national (e.g., UK) and international (AHA, ASE, AEPC) fetal cardiology practice guidelines that also include criteria for personnel competence (e.g., UK). Maintaining fetal cardiology training skills are, however, like previously reported for pediatric cardiologists [10].

Fetal echocardiographic imaging training was mainly provided by observing and performing assessments in our survey. Trainees were mainly assessed by expert review of imaging, case-based discussions, and other workplace-based assessments. Feedback on imaging was provided both during and after imaging and only rarely in other ways. Imaging reports were mainly generated under supervision with feedback provided as appropriate. Independent fetal echocardiography and counseling by core fellows was rare. The variability in studies and abnormal assessments needed in training was large among responses that may reflect differences in learning environment. A recent study showed that skills in fetal echocardiography imaging could effectively be provided also as simulation training [11].

A recent survey from the USA showed that formal education and feedback in counseling techniques is relatively rarely provided for pediatric cardiology core trainees. Observing and performing by trainee with direct observation and feedback by experts was appreciated as the most effective way of learning fetal counseling for cardiac disease in practice. The median number of counseling observed during subspeciality fellowship training was mostly between 50 and 100 [12]. Our survey results agree with this with most respondents advocating the observing and performing teaching approach. Trainees performing counseling was, however, reported by less than half of respondents suggesting an area of improvement in fetal cardiology training. Only a minority of respondents in our survey mentioned didactic teaching or attending formal courses in learning fetal counseling. The number of abnormal cases counseled under supervision was also similar (40–200) in our survey. Although counseling practice is relatively briefly described in fetal cardiology practice guidelines [3, 4], there are more recent reports on predictors and mediators of successful counseling based on parental feedback that could be accounted for in training [13, 14].

As responses were mainly obtained from trained pediatric cardiologists with completed advanced fetal cardiology subspeciality training, the results may not be generalized to trained fetal cardiologist with a background in perinatal or obstetric medicine. However, the coverage of European countries included among respondents indicate relatively good representativeness in Europe overall, but not for other geographical areas. In addition, the survey was also designed to ascertain responses from persons with advanced fetal cardiology training and, thus, with an experience both from obtaining and providing advanced training in the field which adds validity to the results. On the other hand, as trainees were not included among respondents the survey does not include trainee perspective or experience. Respondent center practice details were also included as background factors to generate data related with, e.g., center volume. The results may, nevertheless, include reporting bias and error related with respondent interpretation of the survey questions, although efforts were made to minimize ambiguity in the generation of the electronic questionnaire. Finally, the survey was unable to include how details specified in core and advanced fetal cardiology training, or practice guidelines are addressed and included in training in practice.

In conclusion, there is a marked variation in both core and advanced fetal cardiology training practice in Europe. Although most centers follow current guidelines for practice and training, there seems to be a need for further clarifications of training requirements for advanced fetal cardiology service. The survey results also stress the need to further strengthen fetal cardiology content in core pediatric cardiology training, the need for improved quality assessment of service provided as well as the importance of continued multidisciplinary collaboration within this field.

## Data Availability

Data is available on reasonable request.
